# Development of a low-cost, simple, and rapid identification method for *Glycyrrhiza uralensis* using PCR–RFLP and evaluation of seeds distributed on the market

**DOI:** 10.1007/s11418-025-01950-2

**Published:** 2025-10-06

**Authors:** Tomoyo Nishida, Shinichiro Sawa, Koji Sugimura

**Affiliations:** 1 Global Center for Natural Resources Sciences, Kumamoto University, 5-1, Oe Honmachi, Chuo-Ku, Kumamoto, 862-0973 Japan; 2 International Research Center for Agricultural and Environmental Biology, Kumamoto University, 2-39-1, Kumamoto, Japan

**Keywords:** *Glycyrrhiza uralensis*, PCR–RFLP, Genetic markers, Hybridization, Species identification

## Abstract

Glycyrrhiza is a crude drug used worldwide, especially in China and Japan. *Glycyrrhiza uralensis* Fischer and *Glycyrrhiza glabra* L. are listed as original plants in the 18th revised Japanese Pharmacopoeia; however, *G. uralensis* is considered the most important *Glycyrrhiza* species because it has traditionally been used medicinally. Most *G. uralensis* used in Japan is imported from China, but export restrictions in recent years have raised concerns about stable supply. Therefore, domestic cultivation in Japan is expected to prevent price increases of raw materials and ensure a stable supply. Accurate species identification is an important step in quality control. In recent years, a method for identifying species using molecular markers has been evaluated for the *Glycyrrhiza* genus; however, in this study, we found that a simpler method, PCR–RFLP, using two types of restriction enzymes, can be used to identify *G. uralensis* more cheaply and easily than conventional methods. When applied to the *ITS*1-5.8S-*ITS*2 region, the analysis of seeds distributed in the market showed that only four of the 190 seeds surveyed were *G. uralensis*, while most were *G. glabra*, *G. inflata*, or their hybrids. Therefore, to appropriately use commercially available *G. uralensis* seeds, it is important to correctly identify the species at an early stage. Quick identification using PCR–RFLP is thought to be a useful method for ensuring the adequate supply of *G. uralensis*.

**Graphical Abstract:**

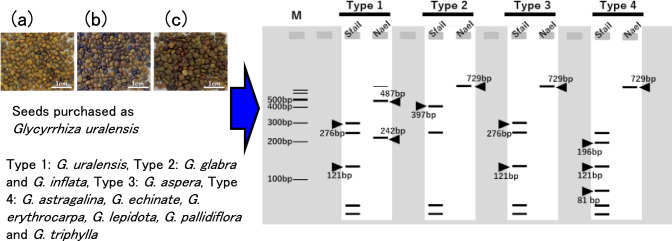

**Supplementary Information:**

The online version contains supplementary material available at 10.1007/s11418-025-01950-2.

## Introduction

Glycyrrhiza is known to have a wide range of medicinal properties, including anti-inflammatory, antioxidant, antibacterial, antiviral, antitumor, hepatoprotective, and neuroprotective effects [1–3], and is one of the most important medicinal plants in the world [4]. *Glycyrrhiza uralensis* Fischer and *Glycyrrhiza glabra* L. are listed in the 18th revised Japanese Pharmacopoeia as the original plants of Glycyrrhiza [5], and *G. uralensis* is traditionally used for medicinal purposes [6]. Glycyrrhiza was included in 214 (72.8%) of 294 formulae for OTC Kampo medicines approved by the Ministry of Health, Labor, and Welfare [7], making it the most consumed crude drug in Japan. However, most Glycyrrhiza are imported from China [8], so it is important to promote their cultivation in Japan.

In *G. uralensis* cultivation, while vegetative propagation using stolons is commonly practiced due to its efficiency, the use of seeds remains important for maintaining genetic diversity and adapting to new cultivation environments. Therefore, combining both methods can be beneficial for the sustainable cultivation of *G*. *uralensis*.

In China, three species are used as source plants of Glycyrrhiza: the two species used in Japan and *Glycyrrhiza inflata* Batalin [9]. The phylogenetic relationships among these three species have been investigated [4, 10, 11]. It has been reported that they have the same chromosome number (2*n* = 16), do not exhibit gametic isolation, and that interspecific hybridization occurs under natural conditions in areas where their habitats overlap [12]. A previous survey reported that fruits were mixed in a warehouse of a wholesale firm in the Xinjiang region, where the distribution ranges overlap [13]. Therefore, accurate species identification is important when seeds are used.

The morphological identification of *Glycyrrhiza* is performed using leaves, inflorescences, pods, and seeds [14]. *G. uralensis*, the most important species for medicinal purposes, is the most heat-sensitive of the three *Glycyrrhiza* species; it grows mainly in desert areas [14]. Therefore, it is difficult for *G. uralensis* to flower and bear fruit stably in areas with high temperatures and heavy rain, such as Kyushu, Japan. Moreover, it is difficult to observe the morphology of its inflorescences and pods, which are important for classification. Furthermore, because there is a large variation in the morphology of hybrid species, it is difficult to accurately identify species in the genus *Glycyrrhiza* based on morphological knowledge alone [15]. *G. uralensis*, *G. glabra*, and *G. inflata* contain species-specific components called glycycoumarin, glabridin, and licochalcone A, respectively [10]. However, the content of these components varies among individuals, and there have been reports of individuals in which specific components were not detected. Furthermore, because only one component is often detected in hybrid individuals, it is difficult to identify species of *Glycyrrhiza* based on these components alone [10].

Recently, a highly accurate method for identifying *Glycyrrhiza* using DNA sequences has been established. It has been shown that *G. uralensis* can be identified by a difference of four bases in the internal transcribed spacer (*ITS*) sequence of the nuclear ribosomal DNA [15]. Although this method offers high reliability and resolution, DNA sequencing requires either access to specialized equipment or reliance on external services, both of which incur significant time and financial resources [16]. Therefore, a cheaper and faster approach is required to identify many samples simultaneously.

PCR–RFLP is a method used to detect the length patterns of DNA fragments amplified via PCR and cut at specific sequences recognized by restriction enzymes. It can easily and inexpensively identify genotypes using a thermal cycler and an agarose gel electrophoresis apparatus, which are less expensive than conventional DNA sequencers [17]. In addition, if one of the alleles has a restriction enzyme site, it is easy to determine whether the genotype is homozygous or heterozygous based on the resulting band pattern [18]. In crude drugs, it has been reported that Atractylodes, which are difficult to identify by morphology and component chemistry and in which hybrid individuals exist, can be identified with high accuracy using PCR–RFLP analysis of the *ITS* region [19, 20]. Since *ITS* sequences are nuclear DNA and are biparentally inherited, it is possible to identify hybrids when interspecific sequence differences exist. Therefore, it is thought to be suitable for identifying the genus Glycyrrhiza, which is prone to interspecific hybridization.

In this study, we aimed to establish a method for identifying *G. uralensis* using polymerase chain reaction-restriction fragment length polymorphisms (PCR–RFLP) alone, enabling more rapid and cost-effective identification compared to conventional methods. This method enables efficient identification of *G. uralensis* compared to conventional sequencing approaches and can contribute to preserving genetic diversity and facilitating the development of new cultivars. Furthermore, the developed technique was used to determine and evaluate the genotypes of *G. uralensis* seeds commercially available in Japan, which have not been investigated to date.

## Materials and methods

### Seed morphological observation and germination test

Since seeds of Glycyrrhiza are not commonly available through general retail outlets in Japan, seeds labeled as *G. uralensis* were obtained via the internet in 2023. Seeds were purchased from two privately owned specialist companies in Japan (Company C1 and C3) and one major overseas nursery company (Company C2). All of the products are also available to consumer. To evaluate seed morphology, seeds were photographed and measured using ImageJ (http://rsb.info.nih.gov/ij/index.html). The major and minor axis lengths were recorded, and the weight of 100 seeds was measured using an analytical balance.

To assess seed viability and obtain seedlings for sequence analysis, we conducted germination tests using a simple hot water treatment, as recommended by C1 for the purchased seeds. Although sulfuric acid treatment has been reported as effective for Glycyrrhiza seed germination, the hot water method is also commonly used in large-scale cultivation [21]. Seeds were treated with hot water at 60 °C and soaked in water at room temperature (20 °C-25 °C) for approximately a week. After soaking, seeds were placed on moist filter paper in 9-cm Petri dishes and incubated at room temperature. Germination rate was calculated about one week after pretreatment by counting the number of seeds with shoots longer than 2 cm.

### Investigation of growth characteristics

One-year-old plants of *G. uralensis*, that were grown from cell tray seedling from *G. uralensis* seeds (C1, C2) in the Kumamoto University Faculty of Pharmaceutical laboratory, potted in black plastic pot, and cultivated in a plastic greenhouse from 2024 to 2025 were used in this growth characteristics study.

The voucher numbers are C1 (0001-24KUM) and C2 (0002-24KUM). Average values of plant height, number of leaves, leaf length, leaf width, petiole length, number of leaflets, leaflet length, leaflet width, number of stems, and stem diameter during the peak growth period of 1-year-old plants were measured (*n* = 3 for each plant variety). The plants were observed and the growth habit, rachis hairs, petiole hairs, leaflet shape, leaflet margin, leaflet tip shape, leaflet surface color and hairs, leaflet underside color and hairs, and stem color and hairs were recorded.

### HPLC analysis of species-specific phenolic compounds

Dried plant materials were powdered using an Absolute Mill ABS-W (Osaka Chemical Co., Ltd., Osaka, Japan). For extraction, 0.25 g of each powdered sample was mixed with 30 mL of 50% ethanol and subjected to shaking extraction at room temperature for 15 min. After centrifugation at 3000 rpm for 5 min, the supernatant was collected. The residue was then re-extracted with 15 mL of 50% ethanol under the same conditions. Supernatants from both extractions were combined and adjusted to a final volume of 50 mL with 50% ethanol. These extracts were used subsequent phenolic compound analysis.

Quantification of phenolic compounds was performed using an Agilent 1260 Infinity III Prime LC system equipped with a photodiode array detector (Agilent Technologies, CA, USA). A Wakopak Wakosil-II 5C18-200 column (4.6 mm I.D. × 250 mm length; FUJIFILM Wako Pure Chemical Corp., Osaka, Japan) was employed for separation. The mobile phase consisted of acetonitrile–water–acetic acid (40:55:5). The column was maintained at 40 °C, with a flow rate of 1.2 mL/min and an injection volume of 20 μL. Detection wavelengths were set at 280 nm for glabridin (GB), and 350 nm for glycycoumarin (GC) and licochalcone A (LA). Each phenolic compound was identified by comparing both the retention time and UV absorption spectra with those of authentic standards.

### Sequencing analysis

To identify sequence variations unique to *G. uralensis*, we conducted preliminary sequencing of eight germinated individuals: four from C1 (Co1_S1–S4), three from C2 (Co2_S1–S3), and one from C3 (Co3_S1). This analysis aimed to detect species-specific SNPs in the *ITS* region to guide the design of a PCR–RFLP assay.

The cetyltrimethylammonium bromide (CTAB) method was used for DNA extraction. Briefly, the samples were frozen in liquid nitrogen and ground at 1300 rpm for 40 s using a Shake Master NEO (Bio Medical Science, Tokyo, Japan). The frozen powder was transferred to 500 μl of CTAB buffer (100 mM Tris–HCl [pH 8.0], 50 mM EDTA [pH 8.0], 1 M NaCl, and 3% CTAB) in a 1.5 ml microcentrifuge tube. The CTAB buffer and frozen powder were mixed well and incubated at 60 °C for 30 min. After incubation, the mixture was mixed with an equal volume of chloroform: isoamyl alcohol (24:1) and gently mixed at room temperature for 15 min. The mixture was then centrifuged at 15,000 rpm for 1 min at 25 °C. Next, 450 μl of the resulting aqueous phase was transferred to a fresh tube, and the DNA was precipitated by adding 300 μl of isopropanol and mixing via inversion. The precipitated DNA was centrifuged at 15,000 rpm for 10 min at 25 °C. The supernatant was decanted carefully, and the pellet was washed with 190 μl of 70% ethanol. Finally, the pellet was dried at room temperature and dissolved in 30 μl of Tris–EDTA buffer (Tris–EDTA Buffer Solution (pH 8.0), Nacalai Tesque, Kyoto, Japan).

Target segments containing the *ITS*1-5.8S-*ITS*2 region were amplified via PCR using the primer pair *ITS*5 (5'- GGA AGT AAA AGT CGT AAC AAG G -3') and *ITS*4 (5'- TCC TCC GCT TAT TGA TAT GC -3') [22]. PCR amplification was conducted in 40 μl reaction volumes containing 100 ng of template DNA, 20 μl of SapphireAmp Fast PCR Master Mix (Takara Bio, Ohtsu, Japan), and 1.6 μM of each primer. The amplification conditions were as follows: an initial denaturation step of 1 min at 94 °C, 45 cycles of 5 s at 98 °C, 5 s at 55 °C and 15 s at 72 °C, followed by a final extension step of 7 min at 72 °C. The PCR products were electrophoresed on a 1.5% agarose gel to confirm the amplification of the DNA fragments. Next, the PCR products were purified using a FastGene Gel/PCR Extraction Kit (Nippon Genetics, Tokyo, Japan), according to the manufacturer’s instructions. The purified DNA fragment was used as a template, and the DNA sequence was confirmed by the DNA Sequencing Division of Eurofins Genomics, Inc. (Tokyo, Japan). The obtained waveform data were processed using BioEdit (http://www.mbio.ncsu.edu/BioEdit/bioedit.html) to determine the base sequences.

### PCR–RFLP analysis

Based on SNPs identified through preliminary sequencing, we developed a PCR–RFLP assay to efficiently genotype a large number of individuals. We focused on the seed lot from C1, which had high germination rates and showed sequence diversity. After hot water pretreatment, seeds were sown in 128-cell trays. One month later, seedlings were transplanted into 9 cm pots, and grown for an additional 3 months before being moved to field conditions. After approximately one year of growth, a total of 190 individuals were sampled for leaf tissue and DNA extraction.

Fresh leaves were washed, approximately 50 mg of leaves were weighed, and stored at –80 °C until use. The leaf samples were then frozen in liquid nitrogen and ground at 2500 rpm for 30 s using a Mini-Beads Shocker (YASUI KIKAI, Osaka, Japan). The frozen powder was extracted for total DNA using a commercial kit (DNeasy Plant Mini Kit; QIAGEN, Hilden, Germany) according to the manufacturer's protocol.

Target segments containing the *ITS*1-5.8S-*ITS*2 region were amplified as described in the sequencing section, with minor modifications. Reactions were conducted in 50 μl volumes containing 50 ng of template DNA, 1.25 U of Takara ExTaq Polymerase (Takara, Shiga, Japan), 5 μl of 10 × ExTaq Buffer (20 mM Mg^2+^ plus), 4 μl of a dNTP mixture (2.5 μM each), and 0.5 μM of each primer. The PCR amplification conditions were as follows: initial denaturation step of 1 min at 98 °C, 45 cycles of 10 s at 98 °C, 30 s at 55 °C, and 1 min at 72 °C, followed by a final extension step of 2 min at 72 °C. The PCR products were purified using QIAquick® PCR Purification Kit (QIAGEN, Hilden, Germany) according to the manufacturer’s instructions.

Based on the DNA sequence information obtained in this study, we surveyed restriction enzymes that recognize mutations in the *ITS* region using NEBcutter™ v3.0 (https://nc3.neb.com/NEBcutter/). The two restriction enzymes selected were *Nae*I (New England Biolabs Japan, Tokyo, Japan) and *Sfa*NI (Takara, Shiga, Japan). *Nae*I restriction reactions were conducted in 10 μl volumes containing 250 ng of template DNA, 1 μl of 10 × L buffer, and 2 U of *Nae*I. After incubation for 1 h at 37 °C, DNA samples were electrophoresed in 2% agarose gels [Agarose S (Nippon Genetics, Tokyo, Japan) dissolved in 0.5 × Tris–acetate-EDTA (TAE) buffer] for approximately 50 min and visualized via staining with 0.5 μg/ml ethidium bromide. Similarly, *Sfa*NI reactions were conducted in 10 μl volumes containing 250 ng of template DNA, 1 μl of 10 × NEBuffer 3.1, and 2 U of *Sfa*NI. After incubation for 1 h at 37 °C, DNA samples were electrophoresed in 3% agarose gels [Agarose 21 (Nippon Genetics) dissolved in 0.5 × TAE buffer] for approximately 60 min and visualized via staining with 0.5 μg/ml ethidium bromide. Gel images were obtained using a UV transilluminator UVA-15 (ASTEC, Fukuoka, Japan). Fluorescence intensity from the *Nae*I digestion was analyzed using ImageJ software. A graph of the signal intensity was created for each lane, and the peak areas were compared.

## Results and discussion

### Characteristics of commercially available G. uralensis seeds

The results of measuring the length, width, and 100-seed weight of seeds from the three companies used in this study are shown in Table 1. The average values per 100 seeds were as follows: for C1, the seed length was 2.959 ± 0.252 mm, width 2.322 ± 0.180 mm, and weight 0.937 ± 0.026 g; for C2, length was 2.776 ± 0.293 mm, width 2.288 ± 0.169 mm, and weight 0.768 ± 0.010 g; and for C3, length was 2.886 ± 0.324 mm, width 2.366 ± 0.271 mm, and weight 0.829 ± 0.024 g.

**Table 1:** Seeds information

Nursery company	Country of sale	Country of origin	Seed length (mm) (*n* = 100)	Seed width (mm) (*n* = 100)	100-seed weight (g) (*n* = 3)	Germination rate (%)
C1	Japan	China	2.959 ± 0.252^1)^	2.322 ± 0.180	0.937 ± 0.026	56.5
C2	Germany	China	2.776 ± 0.293^**2)^	2.288 ± 0.169	0.768 ± 0.010^*^	52.4
C3	Japan	China	2.886 ± 0.324^*^	2.366 ± 0.271^*^	0.829 ± 0.024	6.9

1) Mean ± SD, 2) The statistical significance of the differences between the strain were determined using the Kruskal–Wallis multiple comparison test with Scheffe’s method. *p < 0.05, **p < 0.01

**Fig. 1:**
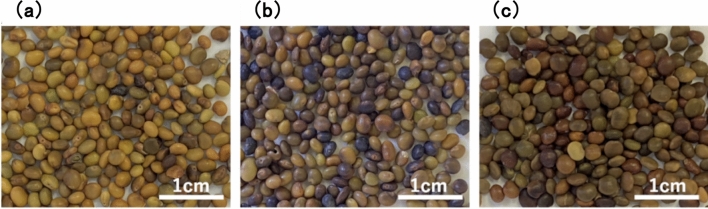
Morphology of commercial *Glycyrrhiza uralensis* seeds. **a** Seeds purchased from Company 1 (C1). **b** Seeds purchased from Company 2 (C2). **c** Seeds purchased from Company 3 (C3)

Statistical analysis revealed significant differences in seed length between C1 and C2 (p < 0.01), and between C2 and C3 (p < 0.05). For seed width, a significant difference was observed between C2 and C3 (p < 0.05), and for seed weight, between C1 and C2 (p < 0.05). These results suggest that seeds from C2 tended to be smaller and lighter than seeds from the other companies. Differences in seed coat color were also noted (Fig. 1a–c). Seeds from C2 exhibited a higher proportion of black seed coats compared to C1 and C3, whereas C3 seeds tended to have darker brown coats than those of C1. Although variations in size, weight, and color were observed among the companies, the large intrasample variability made it difficult to distinguish seed lineage based solely on morphological traits.

To evaluate seed viability and obtain seedlings for sequence analysis, we conducted germination tests using a hot water pretreatment method. Seeds were soaked in hot water at 60 °C and then incubated in water at room temperature for approximately one week, following the protocol provided by Company C1. The germination rates were 56.5% for C1, 52.0% for C2, and 6.9% for C3. While the rates for C1 and C2 were close to the expected value (60%), the markedly low rate in C3 suggests that this hot water treatment was ineffective for C3 seeds. Stronger pretreatments, such as sulfuric acid scarification or mechanical scarification, may be necessary to improve germination in C3 seeds.

These results suggest that although there were notable differences in seed quality and germination capacity among suppliers, the condition of seeds cannot be reliably assessed based solely on external appearance.

### Comparison of growth characteristics of two Glycyrrhiza varieties

**Fig. 2:**
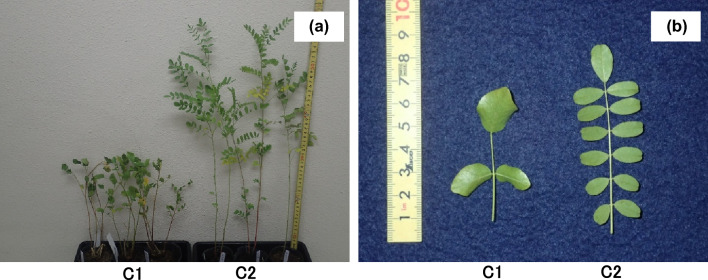
Whole Plant **a** and Leaves **b** of the two varieties (C1, C2) of *Glycyrrhiza uralensis*

Table 2 summarizes the growth characteristics of two Glycyrrhiza varieties (C1, C2) from two companies that had high germination rates after hot seed treatment. The table including the measured plant height (cm), number of leaves, leaf length (cm), leaf width (cm), petiole length (cm), number of leaflets, leaflet length (cm), leaflet width (cm), number of stems, and stem diameter (mm). In addition, this table summarizes the observed results of growth habit, Rachis hairs, Petiole hairs, leaflet shape, leaflet margin, leaflet tip shape, leaflet surface color and hairs, leaflet underside color and hairs, and stem color and hairs. Figure 2a, b shows photographs of whole plant and leaves of C1 and C2.

**Table 2:** Growth characteristics of seedling of the *Glycyrrhiza uralensis*

Category	Characteristics	Plant variety	
		C1	C2
State of a plant	Growth habit	Oblique	Erect
	Plant height(cm)	26.7 ± 2.1	54.7 ± 4.2
Leaf	Number of leaves	9.0 ± 1.0	17.3 ± 2.5
	Leaf length(cm)	5.2 ± 0.8	7.5 ± 0.4
	Leaf width(cm)	3.7 ± 0.2	2.7 ± 0.2
	Petiole length(cm)	2.1 ± 0.3	1.4 ± 0.1
	Rachis hairs	Short hair	Short setae
	Petiole hairs	Short hair	Short setae
Leaflet	Number of leaflets	4.3 ± 1.2	13.0 ± 2.0
	leaflet length(cm)	2.3 ± 0.3	1.5 ± 0.1
	leaflet width(cm)	2.1 ± 0.2	0.8 ± 0.1
	Leaflet shape	Ovate	Oblong
	Leaflet margin	Wavy leaf margin	Flat margin
	Leaflet tip shape	Acute	Retuse
	Leaflet surface color	Slightly glossy green with glandular dots	Dull light green with slightly glandular dots
	Leaflet surface hairs	Hairy on leaf margins	Almost hairless
	Leaflet underside color	Slightly light green	Dull light green with slightly glandular dots
	Leaflet underside hairs	Hairy on veins, slightly hairy on leaf margins	Slightly hairy on veins and leaf margins
Stem	Number of stems	3.7 ± 0.6	2.0 ± 1.0
	Stem diameter(mm)	2.2 ± 0.2	1.9 ± 0.1
	Stem color	The base of the stem is light reddish brown, the rest is green	The base of the stem is red, the rest is green
	Stem hairs	Slightly short hair	Slightly short setae

Data are shown as mean ± SD(*n* = 3 plants per species)

C2 had a greater plant height and leaf length than C1 and had a greater number of leaves and leaflets. The plant habit of C1 was oblique and that of C2 was erect, and the hairs on the rachis and petiole were short haired on C1 and short setae hairs on C2. The shape of the leaflets was ovate in C1 and oblong in C2, the margin of the leaflets was wavy in C1 and flat in C2, and the tips of the leaflets were acute in C1 and retuse in C2. The leaflet surface color of C1 was a slightly glossy green with glandular dots, while C2 was a dull light green with slightly glandular dots. C1 had hairs on the leaflet surface at the leaf margin, while C2 was almost hairless. The leaflet underside color of C1 was slightly light green, while C2 was dull light green with slightly glandular dots. C1 had hairs on the leaflet underside on the veins and leaf margins, while C2 had slightly on the veins and leaf margins. The base of the stem of C1 was reddish-brown, while that of C2 was red. C1 had slightly short hairs on the stem, while C2 had short setae.

Thus, various differences were observed in the growth characteristics of C1 and C2. Among these characteristics, C1 was oblique plant growth, few green ovate leaflets with acute, wavy leaf margins, and glossy surface are consistent with the characteristics of *G. inflata* [23]. On the other hand, the erect plant shape of C2, with many light green leaflets, oblong leaflets that are slightly concave at the tip, have flat leaf margins, and have a dull surface are consistent with the characteristics of *G. glabra* [23]. From the above, it is thought that the leaf morphology characteristics of C1 and C2, among other growth characteristics, are influenced by *G. inflata* for C1 and *G. glabra* for C2.

### HPLC analysis of species-specific phenolic compounds

To further characterize the studied populations, HPLC analysis was conducted to detect species-specific phenolic compounds: glabridin (specific to *G. glabra*), licochalcone A (specific to *G. inflata*), and glycycoumarin (specific to *G. uralensis*).

**Fig. 3:**
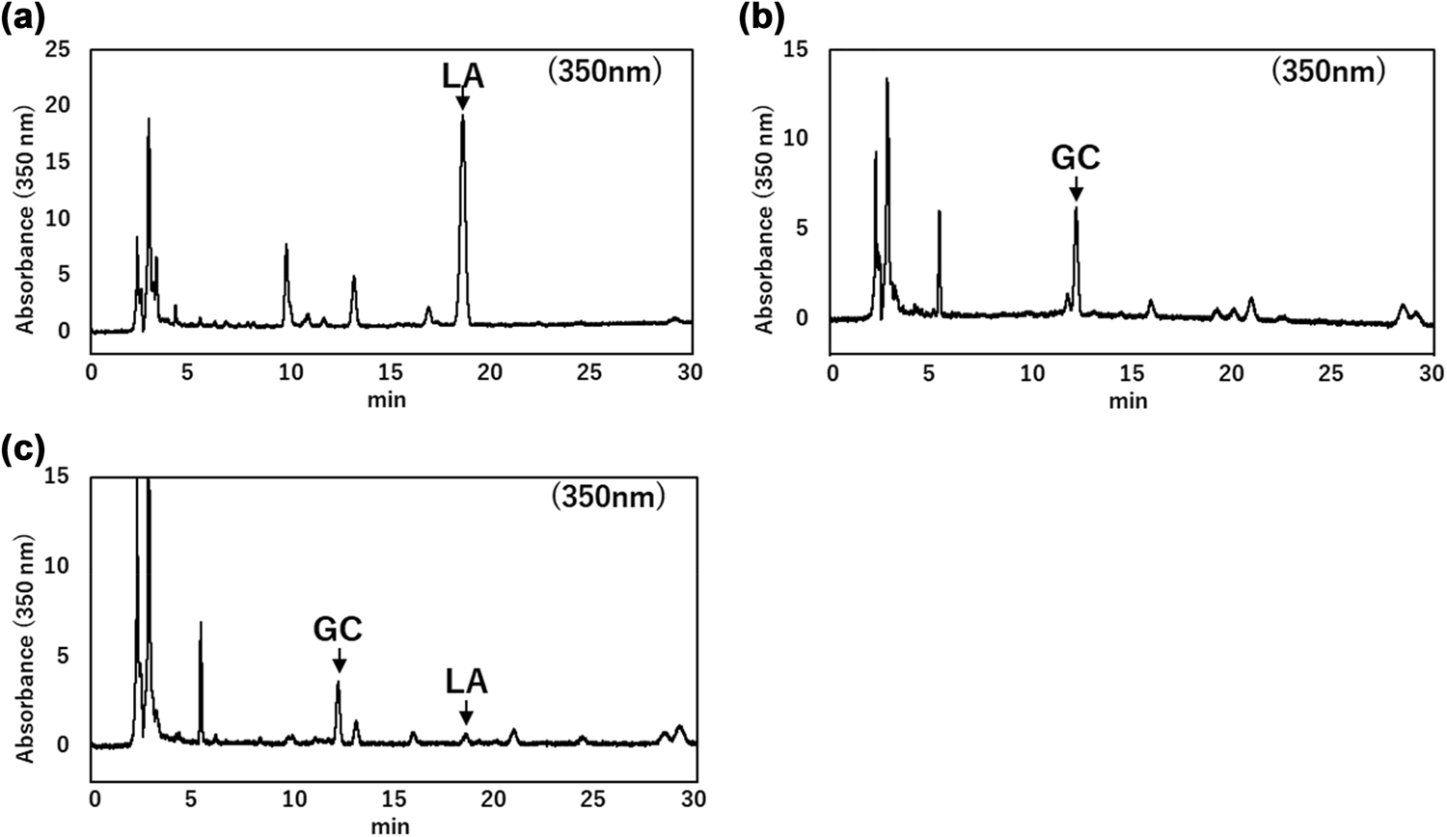
Representative HPLC chromatograms of 50% ethanol extracts of *G. uralensis* roots purchased from C1. Three chromatograms are shown: **a** licochalcone A only, **b** glycycoumarin only, and **c** both compounds. Detection was monitored at 280 nm. LA, licochalcone A; GC, glycycoumarin

In C1, 76 individuals were examined. Three species-specific phenolic compounds were analyzed, but only licochalcone A and glycycoumarin were detected. Four distinct detection patterns for these phenolic compounds were observed: licochalcone A only (*n* = 48), glycycoumarin only (*n* = 12), both licochalcone A and glycycoumarin (*n* = 1), and no detectable phenolic compounds (*n* = 15) (Fig. 3). These results indicate that the C1 seeds may originate from a hybrid population of *G. inflata* and *G. uralensis*.

**Fig. 4:**
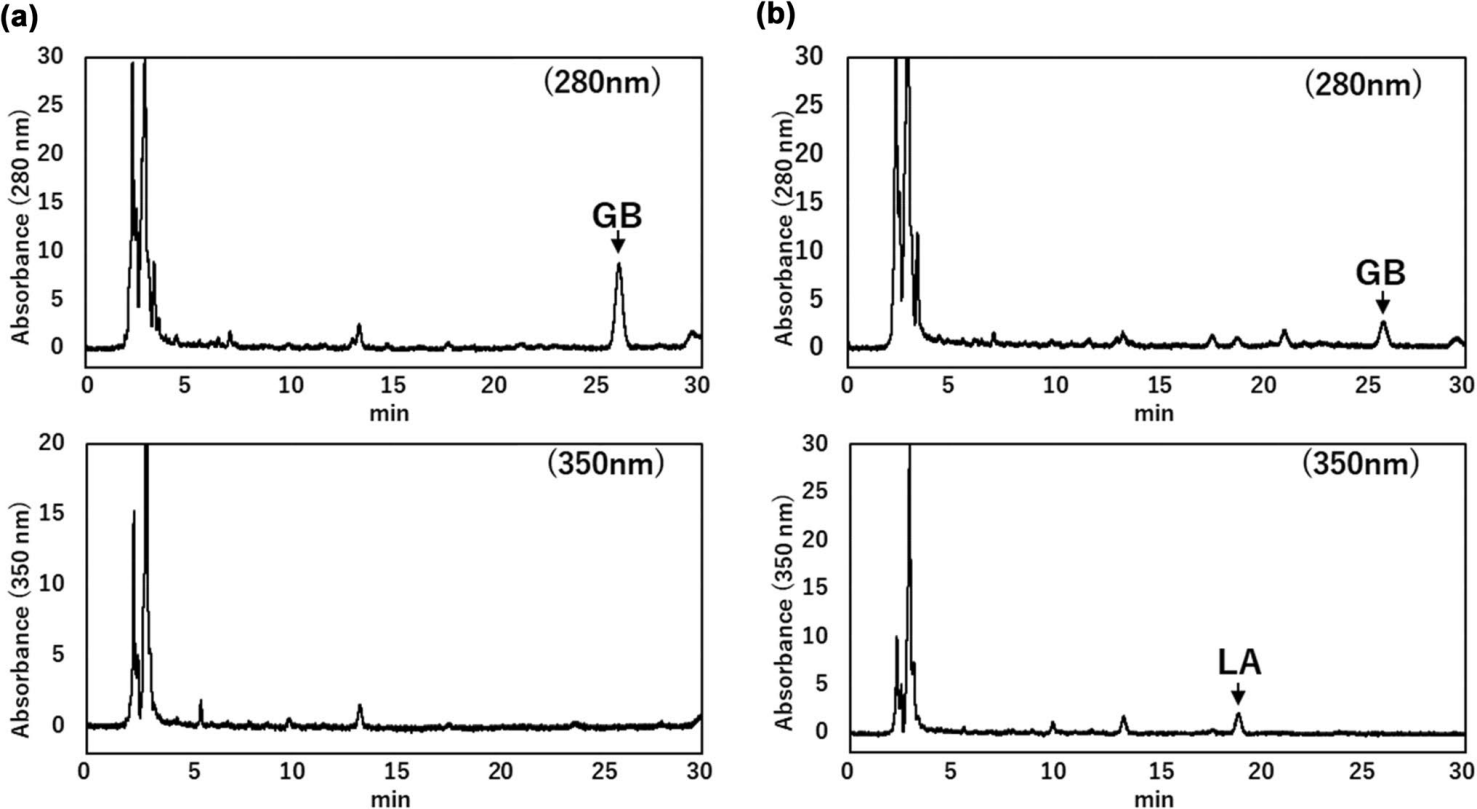
Representative HPLC chromatograms of 50% ethanol extracts of *G. uralensis* roots purchased from C2. Two chromatograms are shown: **a** glabridin only, and **b** glabridin with licochalcone A. Detection was monitored at 350 nm for glabridin and at 280 nm for licochalcone A. GB, glabridin; LA, licochalcone A

In contrast, three representative individuals from C2 were analyzed. All contained glabridin, supporting a strong contribution from *G*. *glabra*. One individual additionally contained licochalcone A, which may indicate potential hybridization with *G*. *inflata*. Glycycoumarin, indicative of *G*. *uralensis*, was not detected in any C2 individuals. Representative chromatograms of C2 (two individuals with glabridin only and one individual with both glabridin and licochalcone A) are shown in Fig. 4.

Collectively, these results suggest that the C1 population is mainly derived from *G. inflata* with possible admixture of *G. uralensis*, whereas the C2 population is primarily derived from *G. glabra* with potential contribution from *G. inflata*. These findings are consistent with the observed morphological characteristics, supporting a mixed origin and potential hybridization in commercially available Glycyrrhiza seeds.

### Comparison of DNA sequences of eight G. uralensis samples

Comparisons of growth characteristics and species-specific phenolic compounds suggested that commercially available *G*. *uralensis* seeds often include individuals hybridized with closely related species. To clarify the genetic composition of these seeds, *ITS* regions were analyzed.

**Fig. 5:**
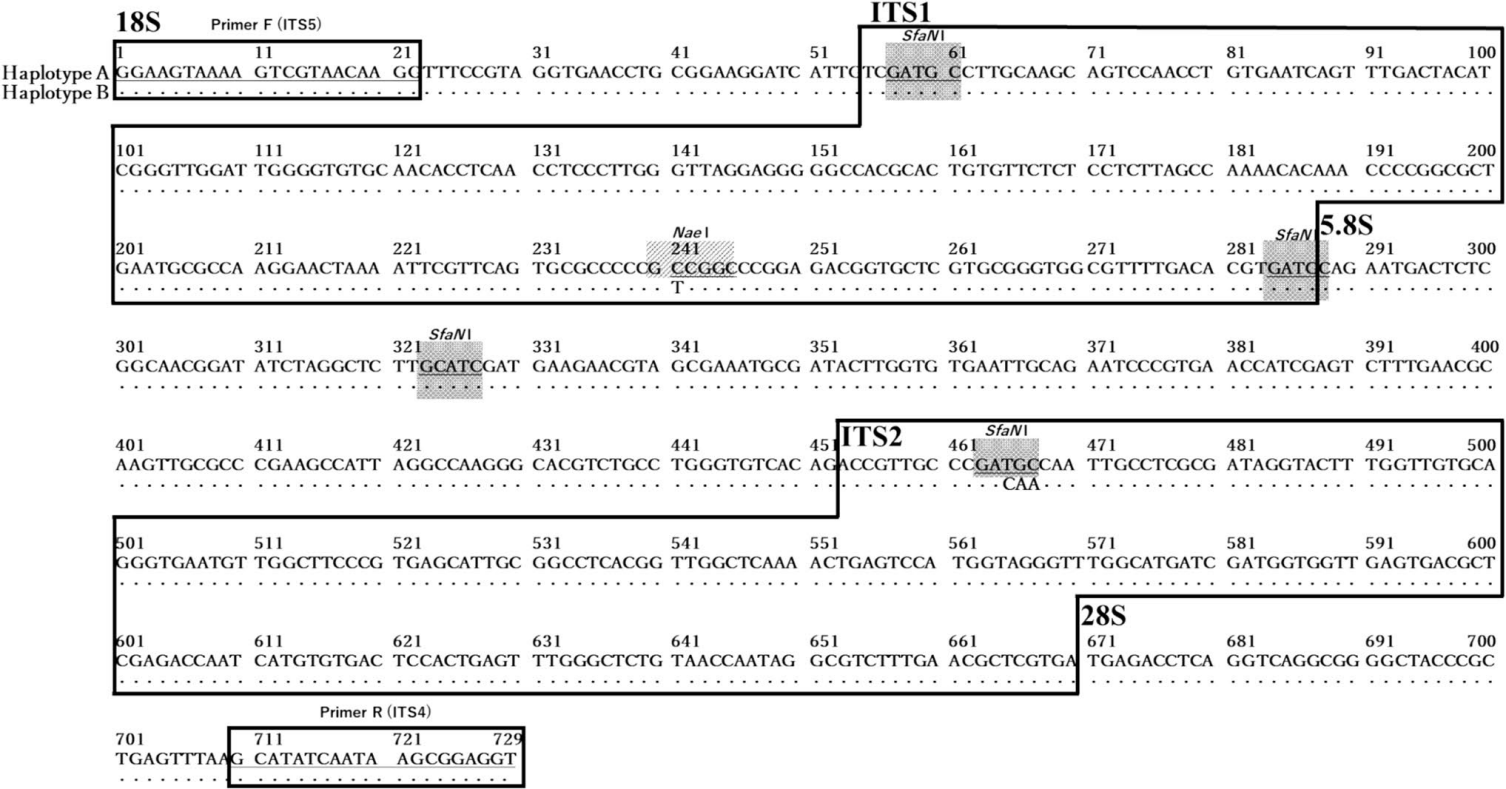
Alignment of the PCR products of Haplotype A (*Glycyrrhiza uralensis*) and Haplotype B (*G. glabra* or *G. inflata*) DNA sequences obtained via sequencing analysis. The alignment consists of the 3′-end of the 18S ribosomal DNA (rDNA) gene (which contains the *ITS*5 primer site), the complete *ITS*1 region, the highly conserved 5.8S rDNA gene sequence, the complete *ITS2* region, and the 5′-end of the 28S rDNA gene (which contains the *ITS*4 primer site). Nucleotides shown in boldface indicate differences between isolates of Haplotype A and Haplotype B. Restriction sites for *Nae*I (GCC ▼ GGC) and *Sfa*NI (GCATC(N)_5_ ▼) are shown with shadows

The DNA sequences of the *ITS* regions of the eight seed samples purchased from the three companies were compared. Owing to differences in one base (position 241) in the *ITS*1 region and three consecutive bases (positions 465–467) in the *ITS2* region, three genotypes were detected: two haplotypes, Haplotype A (C, TGC) and Haplotype B (T, CAA) (Fig. 5), and a heterozygous type with both. The genotypes of each of the eight samples are presented in Table 3. These polymorphisms were consistent with previous reports [15], where Haplotype A was *G. uralensis*, and Haplotype B was *G. glabra* or *G. inflata*.

**Table 3:** Sequence comparison of mutation sites in eight *Glycyrrhiza* samples and haplotypes

Sample ID	Variable site		Haplotype
	241	465–467	
Co1_S1	ADD*	ADD	Hetero
Co1_S2	T	CAA	Haplotype B
Co1_S3	ADD	ADD	Hetero
Co1_S4	T	ADD	Hetero
Co2_S1	ADD	ADD	Hetero
Co2_S2	C	TGC	Haplotype A
Co2_S3	T	CAA	Haplotype B
Co3_S1	T	CAA	Haplotype B

*ADD: Double peaks were observed at this site

**Fig. 6:**
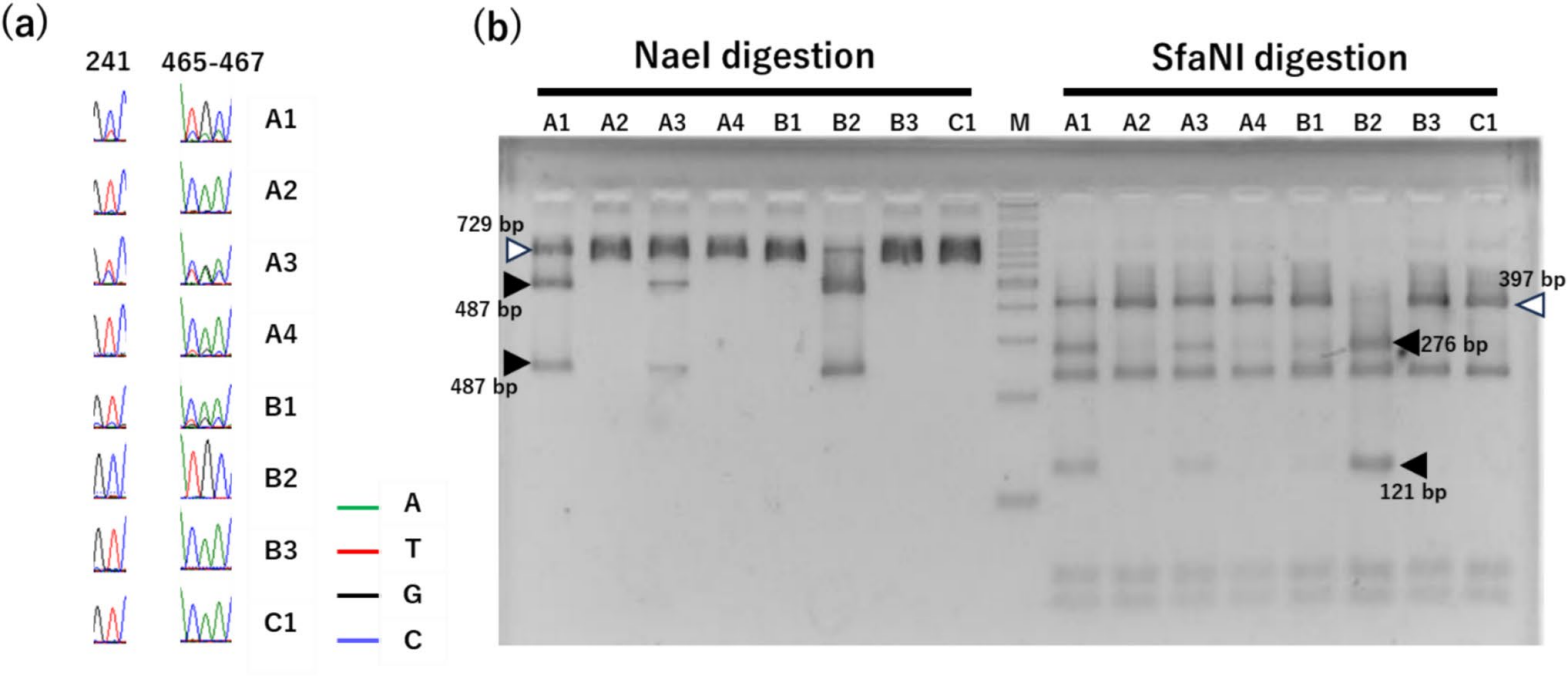
Chromatographic file showing the results of Sanger sequencing and agarose gel electrophoresis after PCR–RFLP. **a** Chromatography file of mutation sites of eight samples of Glycyrrhiza. A is adenine (green), T is thymine (red), G is guanine (black), and C is cytosine (blue). **b** Electrophoresis results of eight Glycyrrhiza samples whose *ITS* sequences were treated with two restriction enzymes, *Nae*I and *Sfa*N I. Lane M is a 100 bp ladder size marker. Black arrows indicate Haplotype A -specific bands. White arrows indicate type Haplotype B-specific bands

The heterozygous genotype, containing both Haplotypes A and B, was the most frequently detected and observed in four samples (Co1_S1, S3, S4, and Co2_S1), accounting for 50% of all samples. Comparing the waveform data of the sequences (Fig. 6**a**), these heterozygous types were divided into three types: A type heterozygous, which has a high proportion of haplotype A; B type heterozygous, which has a high proportion of haplotype B; and even type heterozygous, which has almost the same proportion of the two. Even type heterozygous was seen in Co1_S3, which is thought to be the first filial hybrid of Haplotypes A and B. A type heterozygous was seen in Co1_S1. This suggests that backcrossing events involving Haplotype A may have occurred. B type heterozygous was seen in Co1_S4 and Co2_S1. This suggests that backcrossing by haplotype B, in contrast to A type heterozygous, has occurred. Furthermore, Co1_S4 showed a small subpeak in the *ITS*2 region (positions 465–467) but not in *ITS*1 (position 241), suggesting that the genotype was fixed by continuous backcrossing with haplotype B. The next most common was Haplotype B, representing *G. glabra* or *G. inflata*, and was identified in three samples (Co1_S2, Co2_S3, Co3_S1, 37.5%). Haplotype A was present in only one sample (Co2_S2), indicating that the species was *G. uralensis* (12.5%) as stated on the package.

Comparing by nursery company, C1 had no *G. uralensis* seeds; they only had *G. inflata* or *G. glabra* (25%) or heterozygotes (75%). In C2, *G. uralensis* accounted for 33%, *G. inflata* or *G. glabra* for 33%, and the proportion of heterozygotes was 33%. The seeds obtained from C3 exhibited a low germination rate under the present experimental conditions. Genotypic analysis of one germinated individual revealed that it was either *G. inflata* or *G. glabra.*

These results revealed that the seeds used in this study from the three companies contained not only *G. uralensis* as listed on the package but also many seeds of *G. glabra*, *G. inflata*, and their hybrids. Previous reports on the genotype of *G. uralensis* have been conducted on samples from wild or cultivated areas or dried roots used as raw materials for crude drugs [10–12, 15]. To the best of our knowledge, this is the first report on identifying the genotypes of seeds distributed in Japan. To obtain *G. uralensis* from purchased seeds, it is necessary to identify many individuals, which indicates the need for a cheaper and simpler identification method.

### PCR–RFLP comparison

Based on the *ITS* sequence variations identified above, we developed a PCR–RFLP method to allow easy and cost-effective identification of *G*. *uralensis* seeds. The *ITS* sequences of the eight samples identified via sequencing were digested with the restriction enzymes *Nae*I and *Sfa*NI, as shown in Fig. 6b.

The band pattern of the *ITS* region treated with the restriction enzyme *Nae*I was predicted from sequences obtained via sequence analysis. Because the *Nae*I site is not present in Haplotype B but is present at one site in Haplotype A, it was estimated that a 729-bp uncut band would be detected in Haplotype B, two bands 242 bp and 487 bp long cut at one site would be detected in Haplotype A, and both of these bands would be detected in the heterozygous type.

When the 729-bp DNA fragment amplified via PCR was digested with *Nae*I, one band was detected for Haplotype B (Co1_S2, Co2_S3, and Co3_S1) representing *G. glabra* and *G. inflata*, as expected. However, for Haplotype A (Co2_S2) representing *G. uralensis*, a weak 729-bp band was detected in addition to the two expected bands, 242 bp and 487 bp long. Because *Nae*I is affected by CG-methylase, preliminary tests were conducted in which the *ITS* sequence was amplified via PCR was electrophoresed and then gel-cut before restriction enzyme treatment. The reaction time was changed to 3 h and 16 h, but it was not possible to digest all the bands. The sequences flanking the *NaeI* site were GC-rich, and it is likely that the secondary structure of these regions interfered with enzyme activity. For the heterozygotes, Co1_S4, which only showed a Haplotype B peak in the sequence, and Co2_S1, which had a high proportion of Haplotype B sequences, did not show any cleaved bands and could not be distinguished from Haplotype B. This may be because the proportion of Haplotype A sequences was too low for the cleaved bands to be detected. As expected, three bands of 729, 487, and 242 bp were observed in Co1_S3, in which the ratio of Haplotype A to Haplotype B sequences was almost the same, and in Co1_S1, in which the ratio of Haplotype A sequences was high. Three bands were detected for both Haplotype A and heterozygous types, but the intensity of the cleaved 487 bp and 242 bp bands showed a similar tendency to the peak ratio of Haplotype A sequence in the waveform data of the sequence, suggesting that Haplotype A, which has an uncleaved band remaining, and the heterozygous type can be distinguished by the intensity of the bands. To numerically show the band pattern of Haplotype A, the fluorescence intensity of the bands was analyzed using ImageJ software. It was shown that the fluorescence intensity of the undigested 729-bp band in Haplotype A band pattern was less than 20% of that of the cleaved 487-bp band. These results indicate that the restriction enzyme *Nae*I can be used to identify Haplotype A of *G. uralensis* by analyzing the band patterns.

Next, the band pattern was predicted after the *ITS* region was digested with the restriction enzyme *Sfa*NI, which corresponded to the mutated *ITS2* region. The *Sfa*NI site was estimated to be cleaved at three sites in Haplotype B, giving four bands of 48 bp, 59 bp, 227 bp, and 397 bp; cleaved at four sites in Haplotype A, giving five bands of 48 bp, 59 bp, 121 bp, 227 bp, and 276 bp; six bands of both sites were detected in the heterozygous type. Similar to *Nae*I, the 729-bp DNA fragment amplified via PCR was digested with *Sfa*NI. As expected, four bands were detected for Co1_S2, Co2_S3, and Co3_S1 of Haplotype B, and five bands were detected for B2 of Haplotype A. Although a faint smear-like band was observed near the expected 397 bp size in Haplotype A, it was clear that *Sfa*NI digestion provided a distinct pattern, allowing Haplotype A to be easily distinguished from A type heterozygous without the need for image analysis. The faint band does not affect the overall interpretation of the results, suggesting that *Sfa*NI is still a reliable tool for distinguishing these haplotypes under the current experimental conditions. In the heterozygous type, six bands were detected in Co1_S1 and S3, as expected, where the proportion of Haplotype A sequences was more than half that of the sequence waveform data. For Co1_S4 and Co2_S1, which had a low ratio of Haplotype A sequences, a weak 276 bp band from Haplotype A sequence was detected, in addition to the four bands from Haplotype B sequence. Weak bands from Co1_S4 and Co2_S1 were not detected with *Nae*I, indicating that *Sfa*NI is effective for identifying heterozygotes.

It has been reported that, among the *Glycyrrhiza* genus, the C at position 241 identified using *Nae*I is a sequence characteristic of *G. uralensis*, and the CAA at positions 465–467 identified using *Sfa*NI is a sequence characteristic of *G. glabra* and *G. inflata* [15]. The present results demonstrate that PCR–RFLP using a combination of *Nae*I and *Sfa*NI can efficiently identify Haplotype A *G. uralensis* without the need for DNA sequencing.

### PCR–RFLP analysis of 190 G. uralensis seeds distributed in Japan

To understand the current genotypic status of *G. uralensis* seeds in Japan, we applied the PCR–RFLP method we developed to analyze 190 seeds that we cultivated from a commercial seed lot labeled as *G. uralensis* and purchased from Company C1. This seed lot was selected for detailed analysis because it exhibited a high germination rate and clear sequence diversity in our preliminary tests, making it suitable for large-scale genotyping. The results showed that 59 seeds were of Haplotype B, 127 seeds were heterozygotes, and only 4 seeds were identified as Haplotype A, which corresponds to true *G. uralensis* (Table 4). In addition to Haplotypes A and B (C/T, CAA/TGC or T, CAA/TGC) that could be identified using *Sfa*NI, heterozygotes (C/T, TGC) that could only be identified using *Nae*I were also identified. Since the *ITS2* region only contains the TGC sequence, this heterozygosity does not involve Haplotype B, suggesting a combination of the newly identified Haplotype C (T, TGC) and Haplotype A.

**Table 4:** Haplotypes of commercially available *Glycyrrhiza uralensis*

Variable site		Number	Haplotype	Species
241	465–467			
C	TGC	4	Haplotype A	*G. uralensis*
T	CAA	59	Haplotype B	*G. glabra* or *G. inflata*
C/T or T	CAA/TGC	125	Hetero (Haplotype A × B)	*G. uralensis* ×*G. glabra* or *G. inflata*
C/T	TGC	2	Hetero (Haplotype A × C)	*G. uralensis* × *G. aspera*

**Fig. 7:**
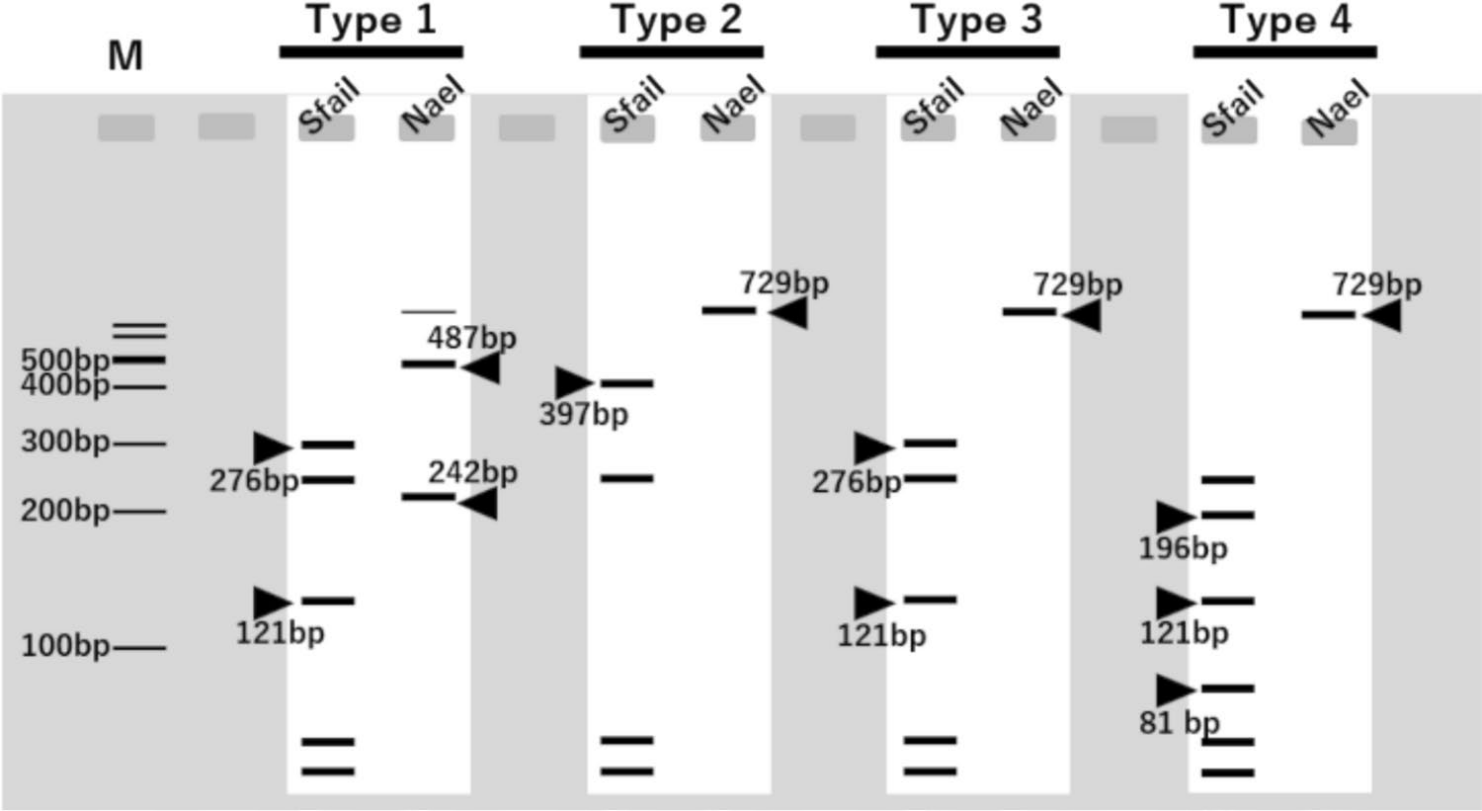
Restriction enzyme cleavage types of *ITS* sequences predicted for 10 species of *Glycyrrhiza. *Type 1: *G. uralensis*, Type 2: *G. glabra* and *G. inflata*, Type 3: *G. aspera*, Type 4: *G. astragalina*, *G. echinate*, *G. erythrocarpa*, *G. lepidota*, *G. pallidiflora*, and *G. triphylla*. Lane M is a 100 bp ladder size marker. The arrows indicate the characteristic bands of each type

To identify hybrid species, the *ITS1*, 5.8S ribosomal RNA, and *ITS2* regions of *Glycyrrhiza* were searched using the National Center for Biotechnology Information database. As a result, we obtained the sequence information for *Glycyrrhiza* aspera Pall. (MT923588) and *Glycyrrhiza astragalina* Gillies ex Hook. & Arn. (GQ246133), *Glycyrrhiza echinata* L. (MT350373), *Glycyrrhiza erythrocarpa* (Vassilcz.) M.N.Abdull. (GQ246131), *Glycyrrhiza lepidota* Pursh (MT350372), *Glycyrrhiza triphylla* Fisch. & C.A. Mey. (OR678310), and *Glycyrrhiza pallidiflora* Maxim. (MT923593). The predicted band patterns of the three species analyzed in this study, along with those of seven *ITS* sequences obtained from the database, are shown in Fig. 7 after digestion with the restriction enzymes *Nae*I and *Sfa*NI. The alignment of Haplotypes A and B with the seven *ITS* sequences used for this analysis is provided in Supplementary Figure S1.

Our analysis revealed that the sequence variations of the seven additional species matched Haplotype C. Furthermore, six of these seven species (excluding *G. aspera*) had an additional *Sfa*NI restriction site, resulting in six cleavage sites. These findings suggest that some seed samples may be hybrids involving *G. aspera* and *G. uralensis*. Notably, hybrid individuals with *G. aspera* as a possible maternal parent have previously been reported in the Xinjiang region [24], and the present findings are consistent with those observations.

These results suggest that some of the seeds labeled as *G. uralensis* may have originated from regions such as Xinjiang, where interspecific hybridization among *Glycyrrhiza* species has been reported. Given the widespread cultivation and seed trade in that area, it is plausible that such hybrid seeds are being mislabeled and distributed as *G. uralensis*. This underscores the need for genetic verification techniques, such as the PCR–RFLP method used here, to ensure accurate species identification and maintain the quality of Glycyrrhiza propagation materials.

In addition to the species for which sequences were obtained from NCBI in this study, three other species have been reported to grow wild in China: *Glycyrrhiza squamulosa* Franchet, *Glycyrrhiza yunnanensis* S. H. Cheng & L. K. Dai ex P. C. Li, and *Glycyrrhiza eglandulosa* X. Y. Li, Bull. Bot. Res. [25]. Future research will involve sequencing and comparing these lesser-known species, which is expected to improve identification accuracy and elucidate the genetic diversity of cultivated and distributed Glycyrrhiza seeds.

From the above results, it was found that of the 190 seeds of *G. uralensis* commercially available in Japan and investigated in this study, only 4 (2.1%) were genotyped as *G. uralensis*; most were seeds of *G. glabra* or *G. inflata* or hybrids of *G. uralensis*, *G. glabra*, *G. inflata*, and *G. aspera*. Hence, it was revealed that there was a large discrepancy between the species names written on the package and their actual contents.

As some individuals were found to hybridize with *G. aspera*, it was important to perform a comprehensive evaluation using two restriction enzymes, *Sfa*NI and *Nae*I. It is thought that *G. uralensis* can be more efficiently identified by first using *Sfa*NI to exclude individuals containing Haplotype B gene, and then performing *Nae*I restriction to confirm that they are indeed of Haplotype A.

## Conclusion

The demand for the crude drug Glycyrrhiza has been increasing annually. However, due to concerns about desertification caused by excessive harvesting, a bill was enacted in China in 2000 to restrict harvesting and require sellers to be registered [6]. As a result, domestic cultivation is expected to prevent the price of raw materials from increasing and ensure a stable supply in Japan. In recent years, Glycyrrhiza grown in Japan has been used as a raw material for crude drugs, but the amount remained small [8]. In addition to the currently used stolon propagation method, there is an increasing need for cultivation tests using genetically diverse seeds to select strains with traits suitable for each region in order to expand the cultivation area. The results of various experiments in this study revealed that there is a high possibility that *G. uralensis* seeds widely distributed in Japan, from major seed companies to privately run specialist businesses, are mixed with other species or hybrids at a fairly high frequency. Based on seed and leaf morphology as well as HPLC analysis of the roots, it was possible to infer the presence of different species and hybrids at the population level; however, DNA analysis was essential for accurately identifying the species of individual plants. Therefore, when using seedlings grown from commonly available *G. uralensis* seeds for trial cultivation to produce crude drugs, it is important to use only seedlings that can be reliably identified as *G. uralensis* by collecting a small number of leaves and performing PCR–RFLP analysis on the *ITS* sequence, which is a newly developed, inexpensive, simple, reliable, and efficient method, before starting full-scale cultivation experiments. This method is considered to be a reliable and effective method, since both leaf morphological observation and root HPLC analysis suggested the possibility of hybridization.

In this paper, we developed a simple method for identifying *G*. *uralensis*, but because the *ITS* region sequences were identical, *G*. *glabra* and *G*. *inflata* could not be distinguished without using HPLC analysis. *G*. *glabra* is not used in herbal medicine, but is listed in the Japan Pharmacopoeia, so it is a commercially important species. It has been reported that *G*. *glabra* and *G*. *inflata* can be identified by the *psbA-trnH* region. However, because the chloroplast genome is maternally inherited in most plant species, this method cannot detect hybrids or heterozygous individuals derived from interspecific crosses, making it unsuitable for comprehensive genetic identification in breeding or propagation programs. In the future, it is expected that a simple identification method using genomic DNA will be developed for these two species.

## Supplementary Information

Below is the link to the electronic supplementary material.

**Additional file 1: **Fig. S1**.** Alignment of the DNA sequences of PCR products from Haplotype A (*Glycyrrhiza uralensis*) and Haplotype B (*G. glabra* or *G. inflata*), along with *ITS* region sequences of seven *Glycyrrhiza* species retrieved from NCBI. The alignment of Haplotypes A and B consists of the 3′-end of the 18S ribosomal DNA (rDNA) gene (which contains the *ITS*5 primer site), the complete *ITS*1 region, the highly conserved 5.8S rDNA gene sequence, the complete *ITS*2 region, and the 5′-end of the 28S rDNA gene (which contains the *ITS*4 primer site). Since some of the NCBI sequences lacked the 18S and/or 28S rDNA regions, only the complete *ITS*1 region, the 5.8S rDNA gene, and the complete *ITS*2 region were included for all sequences. Restriction sites for *Nae*I (GCC ▼ GGC) and *Sfa*NI (GCATC(N)_5_ ▼) are shown with shadows.
