# Supplementary material for: Development of a low-cost, simple, and rapid identification method for Glycyrrhiza uralensis using PCR–RFLP and evaluation of seeds distributed on the market
Source: J Nat Med. 2025 Oct 6;79(6):1419–32. doi: 10.1007/s11418-025-01950-2 (PMC12620321; doi:10.1007/s11418-025-01950-2)
Supplement: Supplementary file 1 — Additional file 1: Fig. S1. Alignment of the DNA sequences of PCR products from Haplotype A (Glycyrrhiza uralensis) and Haplotype B (G. glabra or G. inflata), along with ITS region sequences of seven Glycyrrhiza species retrieved from NCBI. The alignment of Haplotypes A and B consists of the 3′-end of the 18S ribosomal DNA (rDNA) gene (which contains the ITS5 primer site), the complete ITS1 region, the highly conserved 5.8S rDNA gene sequence, the complete ITS2 region, and the 5′-end of the 28S rDNA gene (which contains the ITS4 primer site). Since some of the NCBI sequences lacked the 18S and/or 28S rDNA regions, only the complete ITS1 region, the 5.8S rDNA gene, and the complete ITS2 region were included for all sequences. Restriction sites for NaeI (GCC ▼ GGC) and SfaNI (GCATC(N)5 ▼) are shown with shadows. [file 11418_2025_1950_MOESM1_ESM.docx]

**Supplementary information for**

**Note**

**Development of a low-cost, simple, and rapid identification method for *Glycyrrhiza uralensis* using PCR-RFLP and evaluation of seeds distributed on the market**

Tomoyo Nishida^1^, Shinichiro Sawa^2^, Koji Sugimura^1＊^

^1^ Global Center for Natural Resources Sciences, Kumamoto University, 5-1, Oe Honmachi, Chuo-ku, Kumamoto 862-0973, Japan; sugimura@kumamoto-u.ac.jp (K.S.), neothiyo4649@kumamoto-u.ac.jp (T.N.)

^2^ International Research Center for Agricultural and Environmental Biology, Kumamoto University, 2-39-1 Kumamoto, Japan; sawa@kumamoto-u.ac.jp (S.S.)

* Correspondence: sugimura@kumamoto-u.ac.jp

ORCID 0000-0002-4507-5749, Tel +81-96-371-4878

**Supporting figure (Figure S1)**


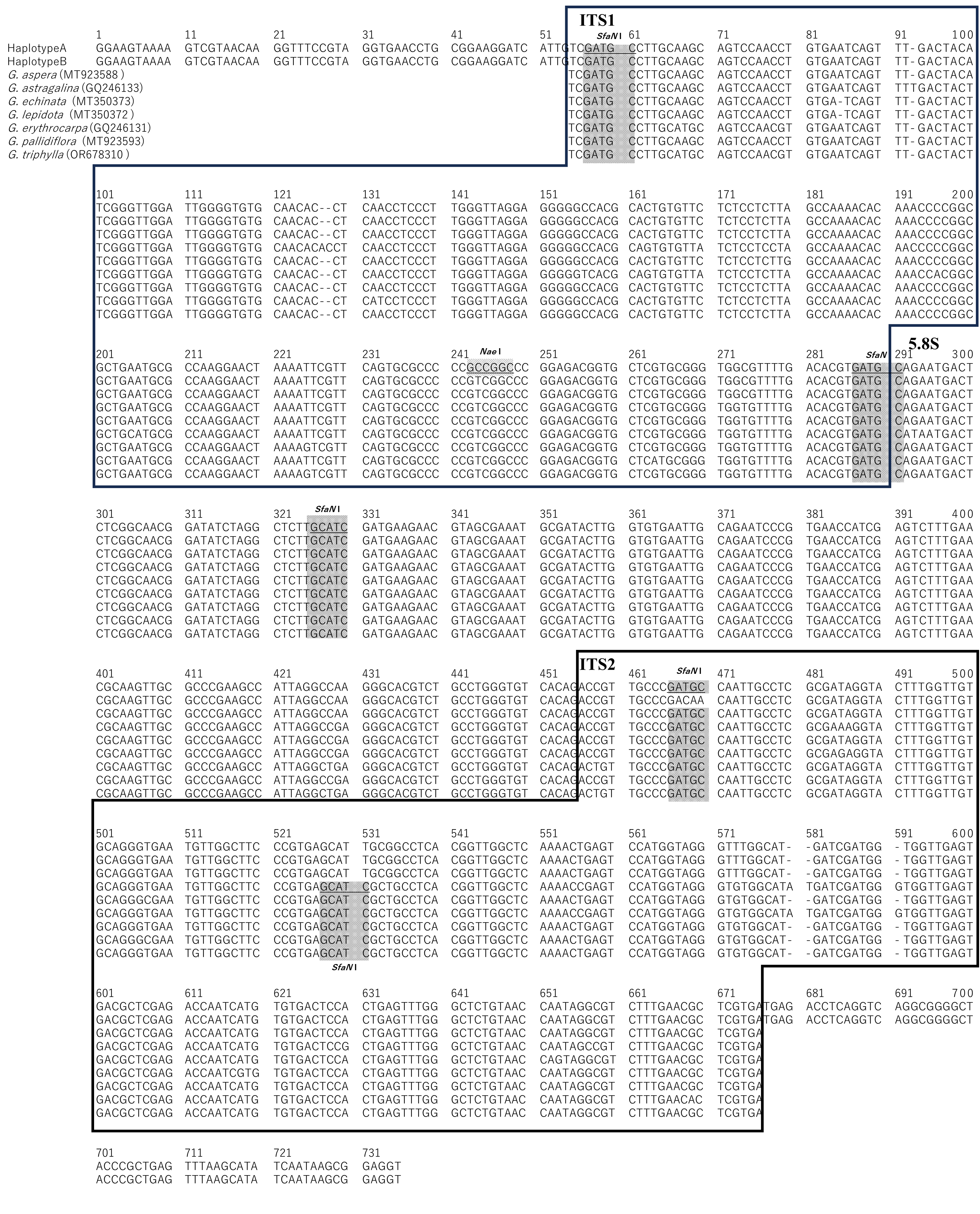


**Fig. S1** Alignment of the DNA sequences of PCR products from Haplotype A (*Glycyrrhiza uralensis*) and Haplotype B (*G. glabra* or *G. inflata*), along with *ITS* region sequences of seven *Glycyrrhiza* species retrieved from NCBI. The alignment of Haplotypes A and B consists of the 3′-end of the 18S ribosomal DNA (rDNA) gene (which contains the *ITS*5 primer site), the complete *ITS*1 region, the highly conserved 5.8S rDNA gene sequence, the complete *ITS*2 region, and the 5′-end of the 28S rDNA gene (which contains the *ITS*4 primer site). Since some of the NCBI sequences lacked the 18S and/or 28S rDNA regions, only the complete *ITS*1 region, the 5.8S rDNA gene, and the complete *ITS*2 region were included for all sequences. Restriction sites for *Nae*I (GCC ▼ GGC) and *Sfa*NI (GCATC(N)_5_ ▼) are shown with shadows.
